# Opinion dynamics with backfire effect and biased assimilation

**DOI:** 10.1371/journal.pone.0256922

**Published:** 2021-09-01

**Authors:** Xi Chen, Panayiotis Tsaparas, Jefrey Lijffijt, Tijl De Bie

**Affiliations:** 1 Department of Electronics and Information Systems, IDLab, Ghent University, Ghent, Belgium; 2 Department of Computer Science and Engineering, University of Ioannina, Ioannina, Greece; Utrecht University, NETHERLANDS

## Abstract

The democratization of AI tools for content generation, combined with unrestricted access to mass media for all (e.g. through microblogging and social media), makes it increasingly hard for people to distinguish fact from fiction. This raises the question of how individual opinions evolve in such a networked environment without grounding in a known reality. The dominant approach to studying this problem uses simple models from the social sciences on how individuals change their opinions when exposed to their social neighborhood, and applies them on large social networks. We propose a novel model that incorporates two known social phenomena: (i) *Biased Assimilation*: the tendency of individuals to adopt other opinions if they are similar to their own; (ii) *Backfire Effect*: the fact that an opposite opinion may further entrench people in their stances, making their opinions more extreme instead of moderating them. To the best of our knowledge, this is the first DeGroot-type opinion formation model that captures the Backfire Effect. A thorough theoretical and empirical analysis of the proposed model reveals intuitive conditions for polarization and consensus to exist, as well as the properties of the resulting opinions.

## Introduction

Recent years have seen an increasing amount of attention from the computational social science in the study of opinion formation and polarization over social networks, with applications ranging from politics to brand perception [1–3]. Much of this research leverages pre-existing opinion formation models that have been studied for decades [4, 5]. These models formalize the fact that people form their opinions through interactions with others. One of the best-known models is the DeGroot model [6], which considers an individual’s opinion as dynamic and updates it iteratively as the weighted average of the individual’s current opinion and those of her social neighbors. The weights represent the strength of the social connections.

The DeGroot model is elegant and intuitive, and it guarantees that the opinions converge towards a consensus [4, 6]. However, opinions formed with it cannot polarize, which contradicts empirical observations [7, 8]. Variants of the DeGroot model have been proposed to incorporate *biased assimilation* [9, 10], which is also known as *confirmation bias* or *myside bias* and refers to the phenomenon where information that corroborates someone’s beliefs affects those beliefs more strongly than information that contradicts them [11]. Incorporating biased assimilation has been shown to potentially lead to polarization [10] or opinion clustering [9].

An extreme manifestation of confirmation bias is a behavior known in social psychology as the *Backfire Effect* [12, 13]. It refers to the fact that, when an individual is faced with information that contradicts her opinion, she will not only tend to discredit it, but will also become more entrenched and thus extreme in her own opinion. The backfire effect may help explain the emergence of polarization. Yet, it has so far been overlooked by existing opinion formation models.

Motivated by these observations, we propose a novel opinion formation model that simultaneously models the Backfire Effect and Biased Assimilation—the BEBA model. BEBA depends on a single—intuitive, node-dependent—parameter *β*_*i*_, which we call the *entrenchment* of node *i*. The parameter captures both the tendency of node *i* to become more entrenched by opposing opinions and the bias towards assimilating opinions favorable to its own. Our main contributions are:

- We propose the BEBA model of opinion formation, which accounts for both the Backfire Effect and Biased Assimilation. To the best of our knowledge BEBA is the first DeGroot-type opinion formation model that incorporates the Backfire Effect.
- We theoretically analyze the BEBA model, studying conditions for reaching consensus or polarization.
- We empirically evaluate, on real and synthetic data, the influence of the entrenchment parameter, the initial opinions, and the network topology, on the opinion dynamics of BEBA.

## Related work

Opinion formation has been studied in diverse research fields, from psychology and social sciences to economics and physics [4, 5]. The former mostly use empirical methods to understand the factors that affect opinion formation, while the latter mostly aim to understand emergent behavior implied by these theories.

Two observations from psychology and social sciences relating to our work are the biased assimilation and backfire effect [14, 15], which state that individuals are more inclined to accept opinions closer to their own [11], and that, when exposed to the opposite opinions, individuals entrench themselves in their own opinions [12, 16, 17], respectively. The existence of the backfire effect is controversial. It is observed in many studies, but there are also failures to find the evidence of it [18, 19]. For example, it is reported negligible on Reddit in a recent study [20]. However, the result may not be robust because the expressed opinions gathered on Reddit are not necessarily consistent with people’s *intrinsic* opinions [21]. The backfire effect remains to be further investigated on improved measures and experimental designs [19], and our modeling of it serves that purpose.

We study the common setting where opinions are formalized as real values, formed through social interactions (see [4] and [5] for surveys). Existing opinion formation models can be described as linear or nonlinear depending on how the opinions are represented [22]. The most popular models include the Voter model [23, 24], the DeGroot model [6], and the Friedkin-Johnsen model [21]. Yet, none of these account for the biased assimilation or backfire effect.

There is work on modeling the fact that users are more influenced by opinions closer to their own. The bounded confidence models [25–27] assume that a user is influenced only by opinions that are within *ϵ* of its own. With rewiring and the relaxation of the bound, the variations of the bounded confidence model are used to further model confirmation bias and polarization in the formation of public opinion [28]. The work of Kempe et al. [29], assumes that there are different types of opinions and users are influenced by opinions of similar types. Das et al. [30], consider a biased version of the voter model that biases individuals to adopt similar opinions.

The work most closely related to ours is that of Dandekar et al. [10], who propose a variant of the DeGroot model to capture the biased assimilation effect. Their model is called the Biased Opinion Formation (BOF) model, and we treat it as our baseline because both ours and the BOF model are DeGroot-type. In the BOF model, the importance that a node attaches to the opinion of a neighbor depends on their agreement. However, it cannot model the backfire effect and introduces cognitive irrationality. We will contrast and highlight the differences between the two models with an illustrative example after formally introducing our model. Before that, the detailed definition of the baseline BOF model, together with that of the vanilla DeGroot model will be introduced in the following section as background of our work.

## Model definition

In this section, we first describe the notations and two existing models that are most relevant to our work (i.e., the DeGroot and the BOF model), then we formally introduce our nonlinear opinion formation model—BEBA, which generalizes the DeGroot model and accounts for both backfire effect and biased assimilation. Finally, we provide a comparison between BEBA and the BOF model on an illustrative example, to contrast and highlight their differences.

### Preliminaries and background

**Notation.** Let *G* = (*V*, *E*) denote a connected undirected network, with *V* = {1, …, *n*} the set of nodes, and *E* ⊆ *V* × *V* the set of *m* = |*E*| edges, where (*i*, *j*) ∈ *E* iff (*j*, *i*) ∈ *E*. When the network is weighted, *w*_*ij*_ = *w*_*ji*_ represents the weight of edge (*i*, *j*). We use *N*(*i*) to denote the set of neighbors of node *i*: *N*(*i*) ≜ {*j* ∈ *V*|(*i*, *j*) ∈ *E*}.

All models we include in this work can be defined as dynamical systems, where opinions are real numbers updated iteratively within a fixed interval of [0, 1] or [−1, 1]. To discriminate between the two intervals, we use *x* for opinions in [0, 1] and *y* for opinions in [−1, 1]. We use *x*_*i*_(*t*) (resp. *y*_*i*_(*t*)) to denote the opinion of node *i* at iteration/time *t* = 0,1,2,…; **x**(*t*) (resp. **y**(*t*)) to denote the opinion vector of the network at time *t*; *x*_*i*_ (resp. *y*_*i*_) to denote the opinion of node *i* after convergence for *t* → ∞ (if that limit exists); and **x** (resp. **y**) to denote the corresponding vector.

**The DeGroot model.** This model [6] is an averaging opinion formation model, where the individual’s opinion is determined by the average of her own opinion and that of her neighbors. More specifically, the updating rule is:

$$\begin{array}{c} x_{i}\left(t+1\right)=\frac{w_{ii}x_{i}\left(t\right)+\sum_{j\in N(i)}w_{ij}x_{j}\left(t\right)}{w_{ii}+\sum_{j\in N(i)}w_{ij}} \end{array}$$ (1)

where *w*_*ii*_ represents the extent to which the node values her own opinion, and *w*_*ij*_ is the strength of the connection/friendship between node *i* and *j*. Iterative opinion updates will converge to a stationary state, where every node has the same opinion *x*_*i*_ = x* [4]. Therefore, the model always reaches consensus, and never polarizes.

**Biased Opinion Formation—BOF.** The BOF model [10] generalizes the DeGroot model to incorporate *biased assimilation*. Given a weighted undirected graph *G* = (*V*, *E*, *w*), every node *i* ∈ *V* is assigned a bias parameter *b*_*i*_ ≥ 0. Higher values of *b*_*i*_ means that node *i* is more biased towards her own opinion. The opinion value *x*_*i*_(*t*) ∈ [0, 1] is interpreted as the degree of support for opinion position 1 (i.e., the highest possible opinion value), while 1 − *x*_*i*_(*t*) is the support for 0. BOF is defined by

$$\begin{array}{c} x_{i}\left(t+1\right)=\frac{w_{ii}x_{i}\left(t\right)+{\left(x_{i}\left(t\right)\right)}^{b_{i}}s_{i}\left(t\right)}{w_{ii}+{\left(x_{i}\left(t\right)\right)}^{b_{i}}s_{i}\left(t\right)+{\left(1-x_{i}\left(t\right)\right)}^{b_{i}}\left(d_{i}-s_{i}\left(t\right)\right)} \end{array}$$ (2)

where *s_i_*(*t*) ≜ ∑_*j* ∈ *N*(*i*)_ *w_ij_x_j_*(*t*) is the weighted sum of *i*’s neighbouring opinions, and *d_i_* ≜ ∑_*j* ∈ *N*(*i*)_ *w_ij_* is the weighted degree of node *i*. During the updating process, node *i* weighs confirming and disconfirming evidence in a biased way: weighing the neighboring support for opinion 1 by ${\left(x_{i}\left(t\right)\right)}^{b_{i}}$, and that for opinion 0 by ${\left(1-x_{i}\left(t\right)\right)}^{b_{i}}$. When *b*_*i*_ = 0, the BOF model is identical to the DeGroot model. However, when *b*_*i*_ ≠ 0, this model introduces cognitive irrationality since an individual’s opinion will change even when the neighboring opinion is the same to its own. We will show that our model does not suffer from this problem.

### The BEBA model

We now define the BEBA model, which also generalizes the DeGroot model to incorporate not only biased assimilation but also the backfire effect. To capture both phenomena, we adapt the DeGroot model by dynamically setting the edge weights. For BEBA, the opinion vector at time *t* is **y**(*t*), with *y*_*i*_(*t*) ∈ [−1, 1]. Rather than using fixed weights as in the DeGroot model, we propose to let the weights be determined by the opinions. Specifically, for an edge (*i*, *j*) ∈ *E* we define the edge weight *w*_*ij*_(*t*) at time *t* as

$$\begin{array}{c} w_{ij}\left(t\right)=\beta_{i}y_{i}\left(t\right)y_{j}\left(t\right)+1. \end{array}$$ (3)

The product *y*_*i*_(*t*)*y*_*j*_(*t*) captures the degree of (dis)agreement between the opinions of node pair (*i*, *j*). The parameter *β*_*i*_ > 0, which we call the *entrenchment parameter* of node *i*, determines the level of the influence caused by that (dis)agreement with node *j* on *i*’s updating with *w*_*ij*_(*t*): the larger, the stronger the biased assimilation and backfire effect.

Given the weights *w*_*ij*_(*t*), the opinions in the BEBA model are updated similarly to the DeGroot model:

$$\begin{array}{c} y_{i}\left(t+1\right)=\frac{w_{ii}y_{i}\left(t\right)+\sum_{j\in N(i)}w_{ij}\left(t\right)y_{j}\left(t\right)}{w_{ii}+\sum_{j\in N(i)}w_{ij}\left(t\right)} \end{array}$$ (4)

Note that when *β*_*i*_ = 0, the BEBA updating rule is identical to that of the DeGroot model (Eq (1)) for unweighted networks. When *β*_*i*_ ≠ 0, we discriminate two cases depending on *w*_*ij*_(*t*):

1. **Backfire Effect is modeled when *w*_*ij*_(*t*) < 0.** We consider two cases: Negative weight means *β*_*i*_ *y*_*i*_(*t*)*y*_*j*_(*t*) < −1. Since *β*_*i*_ > 0, *y*_*i*_(*t*)*y*_*j*_(*t*) < 0, that is, nodes *i* and *j* hold opposing views. Multiplying *y*_*j*_(*t*) with this negative weight *w*_*ij*_(*t*) in the summation in the numerator leads to a contribution of the same sign as *y*_*i*_(*t*), while adding the negative weight to the denominator reduces it, inflating the resulting quotient. The combination of these two effects models the backfire effect.
2. **Biased Assimilation is modeled when *w*_*ij*_(*t*)>0.**
  (a) −1 < *β*_*i*_ *y*_*i*_(*t*)*y*_*j*_(*t*)<0: Here nodes *i* and *j* hold opposing but not too different opinions. Node *i* critically evaluates the conflicting opinion of node *j*, but still assimilates it to a reduced extent.
  (b) 0 < *β*_*i*_ *y*_*i*_(*t*)*y*_*j*_(*t*): Since *β*_*i*_ > 0, node *i* and *j* have both positive or negative opinions here, resulting in an increased weight *w*_*ij*_(*t*). In this case, node *i* assimilates the opinion of neighbor *j* more strongly if the extent of their agreement is stronger.

Note that the denominator in Eq (4) can become 0 resulting in a diverging opinion, or negative causing an unnatural opinion reversal. We consider this situation to be beyond the model’s validity region, and thus we refine the BEBA updating rule as follows:

$$\begin{array}{c} y_{i}\left(t+1\right)=\{\begin{array}{cc} \text{sgn}(y_{i}\left(t\right)) & \text{if}\;w_{ii}+\sum_{j\in N(i)}w_{ij}\left(t\right)\leq 0,\\ \frac{w_{ii}y_{i}\left(t\right)+\sum_{j\in N(i)}w_{ij}\left(t\right)y_{j}\left(t\right)}{w_{ii}+\sum_{j\in N(i)}w_{ij}\left(t\right)} & \text{otherwise}. \end{array} \end{array}$$ (5)

Moreover, for a small denominator, the resulting opinions may fall outside the range [−1, 1]. To address this, we additionally clip negative and positive values at −1 and 1.

### Comparison between BEBA and BOF

There is a similarity between the BOF and our BEBA model, in that both alter the weights in the DeGroot model. Comparing to the linear DeGroot model, both BEBA and BOF are nonlinear. Now we study how the two nonlinear models differ with an illustrative example. Using a star graph consisting of five nodes, we update the opinion of the center node (i.e., node 1) with both models for one iteration and observe how the resulting opinions for the two models differ.

First, we deal with the fact that BOF assumes only positive opinion values, while our model assumes opinions being both positive and negative. Note that the value range of opinions is important in both models, since the BOF model weights the opinion values, while our model exploits the disagreement in the sign. To compare the models, we assume positive opinion *x*_*i*_(*t*) ∈ [0, 1] on all nodes for the update of BOF; and we transform them to [−1, 1] by setting *y*_*i*_(*t*) = 2*x*_*i*_(*t*) − 1 for BEBA. After computing *y*_1_(*t* + 1) with BEBA, we rescale the opinions back to [0, 1].

**Fig 1:**
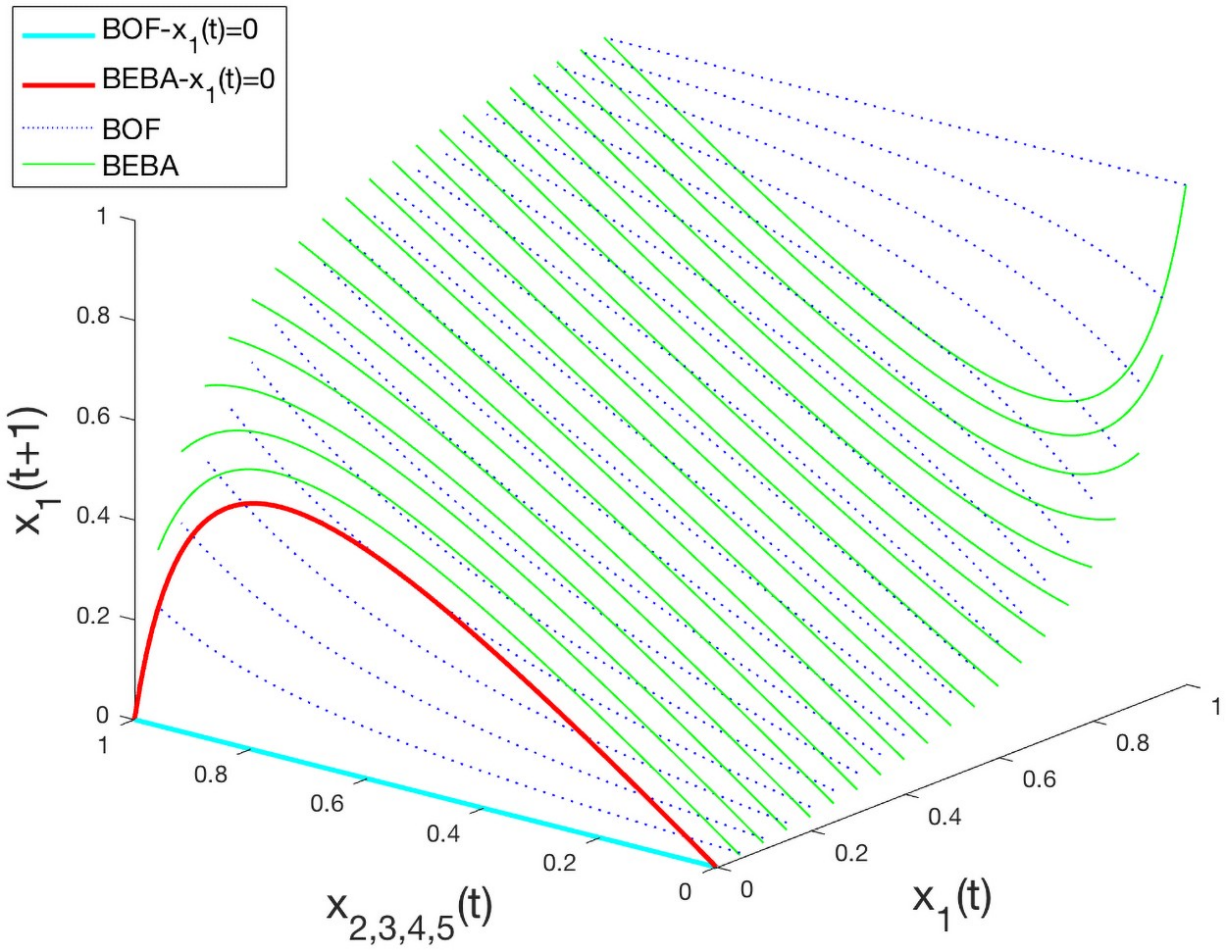
Opinion formation on the star graph.

In this experiment we assume *x*_*i*_(*t*) identical for nodes *i* = 2, 3, 4, 5, and *x*_*i*_(*t*) ∈ [0, 1] for all nodes. We set *w*_11_ = 1 for both models, *b*_1_ = 1 for BOF, and consider the values of 1 and 2.5 for *β*_1_ in BEBA. The opinion value *x*_1_(*t* + 1) updated with both models, as a function of *x*_2,3,4,5_(*t*) and *x*_1_(*t*) is shown in Fig 1. The difference between the two models becomes clearer when *x*_1_(*t*) takes extreme values (i.e., 0 or 1), and we study this below.

**Fig 2:**
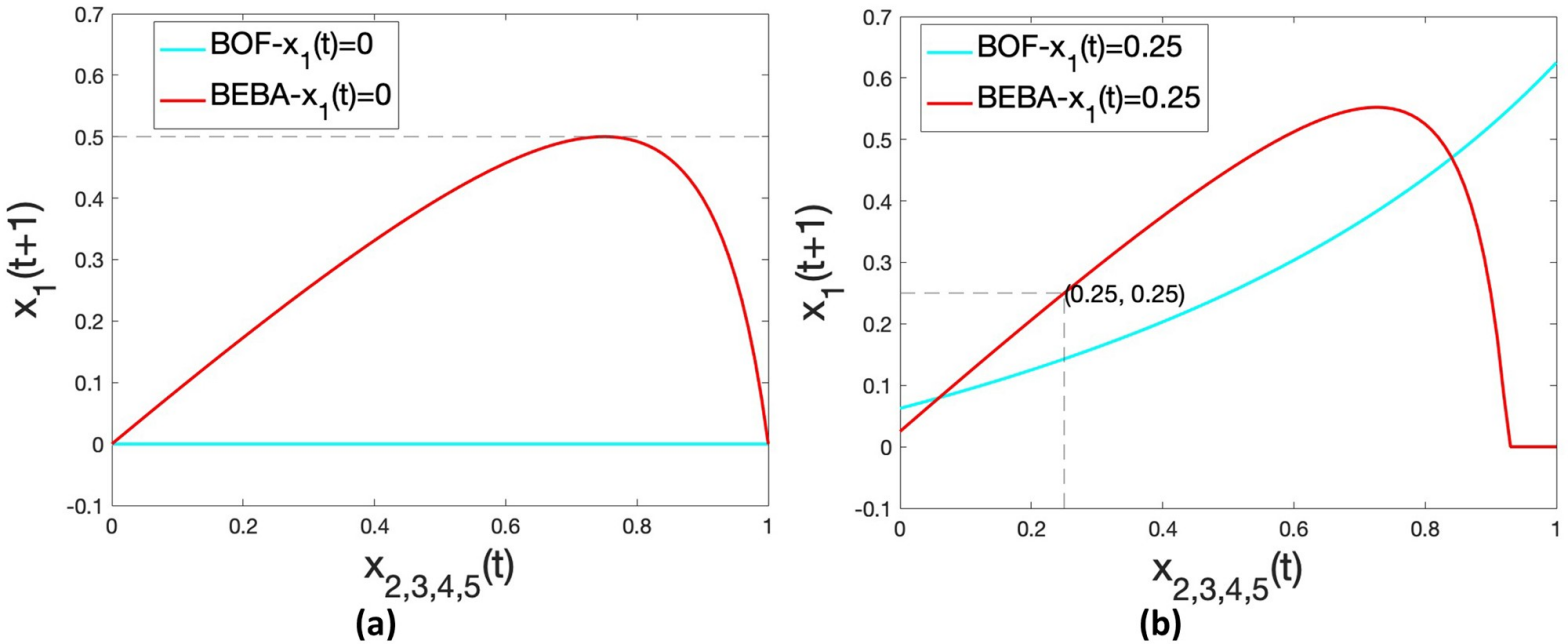
*x*_1_(*t* + 1) as a function of *x*_2,3,4,5_(*t*). (a) *β*_1_ = 1, *b*_1_ = 1, *x*_1_(*t*) = 0; (b) *β*_1_ = 2.5, *b*_1_ = 1, *x*_1_(*t*) = 0.25.

Fig 2(a) shows the curves for the two models when *x*_1_(*t*) = 0. In BOF, the opinion *x*_1_(*t* + 1) remains unchanged at value 0. This is true regardless of the value of *b*_1_. Thus, extreme nodes never change their opinions, even a little, even when they are not biased at all. However, according to biased assimilation, unbiased individuals are influenced by similar opinions, and even extreme nodes assimilate opinions that are close to their own. In contrast, our model better captures the biased assimilation in this case. In Fig 2(a), for *β*_1_ = 1, which corresponds to a mildly biased node, the opinion of node 1 can be moderated by that of her neighbors to different extents, while *x*_1_(*t* + 1) never exceeds 0.5. Therefore, extreme nodes are not stuck in the extremes.

To further highlight the difference between the two models and better understand the backfire effect, we increase *β*_1_ to 2.5, and set *x*_1_(*t*) = 0.25 as shown in Fig 2(b). In BOF, *x*_1_(*t* + 1) becomes smaller than *x*_1_(*t*) = 0.25 even when all neighbors are holding the same opinion *x*_2,3,4,5_(*t*) = 0.25, which does not make sense according to [13]. But in BEBA, we make sure that node 1 does not react to persuasion that coincides with its own current opinion, see point (0.25, 0.25). Meanwhile, we observe the backfire effect with BEBA that when the disagreement between node 1 and her neighbors becomes large (i.e., when *x*_2,3,4,5_(*t*) > 0.9), *x*_1_(*t* + 1) drops under 0.25, until it takes the extreme at opinion 0.

From the plots in Fig 2 we also observe that for the different combinations of *β*_1_ and *x*_1_(*t*), there exists a value of the neighboring opinions that causes the largest change in *x*_1_(*t* + 1). For example, when *β*_1_ = 1 and *x*_1_(*t*) = 0, neighboring opinion of around 0.75 is the most influential as shown in Fig 2(a); for *β*_1_ = 2.5 and *x*_1_(*t*) = 0.25, opinion around 0.7 is the most influential according to Fig 2(b). This provides insight on influence maximization and misinformation correction that a moderate opinion could be more effective than an extreme one.

## Theoretical analysis

This section contains theoretical analysis of the BEBA model for two settings. First we investigate the dynamics of opinions for a single agent in a fixed environment, and secondly we study the dynamics of polarization for all nodes in a connected social network.

### A single agent in a fixed environment

Here we theoretically analyze the limit behavior of a single agent’s opinion in an environment with a fixed opinion. An analysis of this type has been done for the BOF model. The setup is admittedly somewhat artificial but helps to gain a better understanding of BEBA. It has been deemed realistic in cases where the fixed environment consists of the news media, billboards, etc [10]. It also models the situation where the single agent is connected to a network that is large enough such that adding that agent will not meaningfully affect the network.

For the agent *i*, we denote *y*(*t*) ∈ [−1, 1] its opinion at time *t*, *β* > 0 its entrenchment parameter, and *y* its converged opinion—lim_*t* → ∞_ *y*(*t*). We assume the agent weighs its own opinion with *w*_*ii*_ = *w*. For simplicity, we only consider the situation where the environment contains one node, but it should be noted that the analysis below can be easily generalized to several nodes (see S1 Appendix). Let *p* ∈ [−1, 1] be the fixed environmental opinion. Then, according to BEBA, the agent updates its opinion as:

$$\begin{array}{c} y\left(t+1\right)=\{\begin{array}{cc} \text{sgn}(y(t)) & \text{if}\;w+\beta py(t)+1\leq 0,\\ \frac{wy\left(t\right)+\beta p^{2}y\left(t\right)+p}{w+\beta py(t)+1} & \text{otherwise}. \end{array} \end{array}$$ (6)

Before stating a theorem that quantitatively characterizes the limit *y*, we consider the behavior in two cases. The first case is for a sufficiently small *β* (i.e., not biased), while the second is for a sufficiently large *β* (i.e., biased). In the first case, the fixed environment’s opinion *p* will be sufficiently attracting such that *y* = *p* regardless of *y*(*t*). The same is true when *p* = 0: the neutral opinion is never polarizing and thus always attracting. The second case can further be divided into three sub-cases as the limit *y* will depend on the similarity between *y*(*t*) and the environment’s opinion *p*: (a) if *y*(*t*) is similar to *p*, *p* should have an attracting effect on *y*(*t*) such that *y* = *p*; (b) if *y*(*t*) is very different from *p*, however, the backfire effect will cause the agent’s opinion to diverge from *p*, such that *y* = *sgn*(*y*(*t*)); (c) between the former two sub-cases there will be a ‘sweet spot’ where *y*(*t*) is neither sufficiently similar to *p* for *y*(*t*) to converge to *p*, nor sufficiently different for it to diverge to *sgn*(*y*(*t*))—this is an unstable equilibrium where *y*(*t*) remains constant through time, i.e., *y* = *y*(*t*).

This intuition is formalized in the following theorem (proofs in S1 Appendix). For conciseness and transparency, we state it for the situation where *p* ≤ 0 as it is trivial to adapt the theorem for *p* ≥ 0.

**Fig 3:**
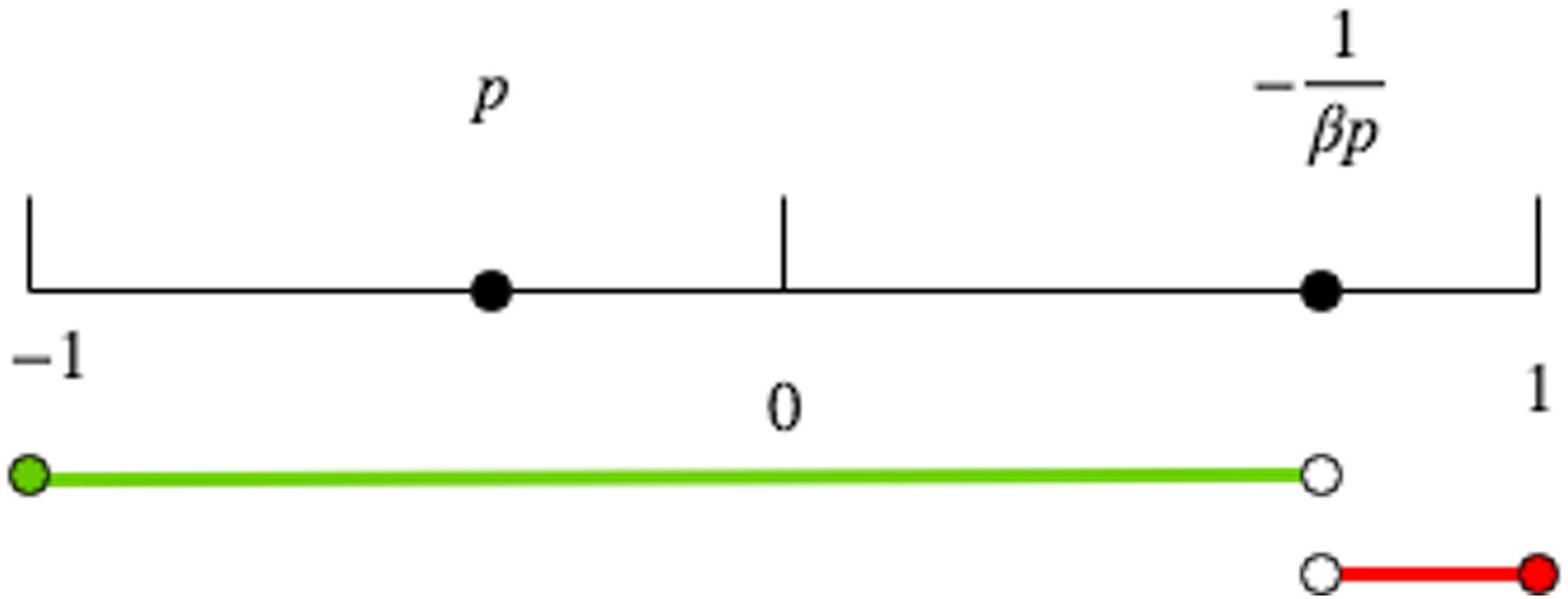
Graphical illustration of Case 2 from Theorem 1 (i.e. *p* < 0 and *β* ≥ −1/*p*). (a) For values of *y*(*t*) in the green range, *y*(*t*) will converge to *y* = *p*. (b) For values of *y*(*t*) in the red range, *y*(*t*) will diverge to *y* = 1. (c) For $y\left(t\right)=-\frac{1}{\beta p}$, *y*(*t*) will not change such that $y=-\frac{1}{\beta p}$.

**Theorem 1**. *For a single agent with opinion y*(*t*) *and entrenchment parameter β in a fixed environment represented by opinion p*, *depending on the value of β relative to p*:

**Case 1:***When p* = 0 or *β* < −1/*p*, *the agent’s opinion always converges to p*: *y* = *p*.
**Case 2:***When p* < 0 *and β* ≥ −1/*p*, *there are three possibilities depending on how similar y*(*t*) *is to p*, *as illustrated in* Fig 3.
  **a:***If*$y\left(t\right)<-\frac{1}{\beta p}$, *y*(*t*) *will be sufficiently attracted to p such that y* = *p*.
  **b:***If*$y\left(t\right)>-\frac{1}{\beta p}$, *y*(*t*) *will diverge away from p such that y* = *sgn*(*y*(*t*)) = 1.
  **c:***If*$y\left(t\right)=-\frac{1}{\beta p}$, *y*(*t*) *will remain constant through time such that*$y=-\frac{1}{\beta p}$.

Theorem 1 already suggests that opinions under the BEBA model evolve to one of three possible states: consensus as in Case 1 and Case 2(a), polarization as in Case 2(b), and an unstable state of persistent disagreement as in Case 2(c).

### Polarization and consensus for all nodes in a network

We now extend our analysis from the single agent to a group of individuals that can update their opinions at any time step *t*. The dynamics of polarization are investigated theoretically with respect to different values of the entrenchment parameter *β*. It was argued by the authors of the BOF model that homophily alone, without biased assimilation was not sufficient for polarization in the DeGroot model [10]. As for BEBA, the backfire effect and biased assimilation are sufficient to lead to polarization or consensus, depending on the parameters and the initial opinions, even when there is no homophily. The theorem below (proofs in S2 Appendix) makes this clear, by providing easy-to-realize sufficient conditions for polarization or consensus to occur.

**Theorem 2**. *Let G* = (*V*, *E*) *be a connected unweighted undirected network. For all i* ∈ *V*, *let y*_*i*_(*t*) ∈ [−1, 0) ∪ (0, 1] *be the opinion of node i at time t*, *w*_*ii*_ = 1 *and* *β*_*i*_ = *β* > 0 *for all i* ∈ *V*. *Denote* **y**(*t*) *the opinion vector of G at time t*, |**y**(*t*)| *the vector with the absolute values of all opinions. Then at convergence the BEBA model can lead to the following states*:

1. *Polarization: When*$\beta >\frac{1}{{\left[\text{min}|y(0)|\right]}^{2}}$, ∀*i* ∈ *V*, |*y*_*i*_| = 1 *and there exist both opinion* −1 *and* 1.
2. *Consensus: When*$\beta <\frac{1}{{\left[\text{max}|y(0)|\right]}^{2}}$, there exists a unique y* ∈ [−*max*|**y**(0)|, *max*|**y**(0)|] *such that* ∀*i* ∈ *V*, *y*_*i*_ = y*.

A special case of particular theoretical interest is when *min*|**y**(0)| = *max*|**y**(0)|. Then there are only two different opinions in the network, with the same absolute value but opposite signs (i.e. they could represent ‘for’ and ‘against’ an issue of interest). In this case, a borderline situation emerges to which we refer as *persistent disagreement*. It can be proved concisely by relying on Theorem 2, and thus we state it as a Corollary:

**Corollary 1**. *Let G* = (*V*, *E*) *be a connected unweighted undirected network where V* = *V*_1_ ∪ *V*_2_, *V*_1_ ∩ *V*_2_ = ∅. *For all i* ∈ *V*, *let w*_*ii*_ = 1 *and β*_*i*_ = *β* > 0. *Assume for all i* ∈ *V*_1_, *y*_*i*_(0) = *y*_0_ *and for all i* ∈ *V*_2_, *y*_*i*_(0) = −*y*_0_ *for some* 0 < *y*_0_ < 1. *Then the BEBA model can result in the following states*:

1. *Polarization: When*$\beta >\frac{1}{y_{0}^{2}}$, ∀*i* ∈ *V*, |*y*_*i*_| = 1 *and there exist both opinion* −1 *and* 1.
2. *Persistent disagreement: When*$\beta =\frac{1}{y_{0}^{2}}$, ∀*i* ∈ *V*_1_, *y*_*i*_(*t*′) = *y*_0_ *and* ∀*i* ∈ *V*_2_, *y*_*i*_(*t*′) = −*y*_0_, *for all* *t*′ ≥ 0.
3. *Consensus: When*$\beta <\frac{1}{y_{0}^{2}}$, there exists a uniquey* ∈ (−*y*_0_, *y*_0_) *such that* ∀*i* ∈ *V*, *y*_*i*_ = y*.

Intriguingly, these conditions in Theorem 2 and Corollary 1 are independent of the network structure and depend only on the entrenchment parameter *β* and the opinion vector at time 0. Yet, it should be noted that the value of the consensus and the eventual polarized state do depend on the network structure. Moreover, the network structure, and the distribution of the opinions over it, do determine whether polarization or consensus will arise when neither of the sufficient conditions of Theorem 2 are satisfied. These claims are confirmed in experiments in the next section.

## Experimental analysis

In the previous section, we provided sufficient conditions for our model to reach consensus or polarization. We now perform an experimental analysis of how these two phenomena manifest themselves on real and synthetic data. Our goal is to answer the following questions:

- In the case when consensus is reached, what is the value of the consensus opinion, and how does the entrenchment parameter *β*, the initial opinions **y**(0), and the network structure affect this value?
- In the case when the opinions polarize, what is the state of the polarization, and how is it affected by the entrenchment parameter *β*, the initial opinions **y**(0), and the network structure?

We use both real-world and synthetic data in our experiments. The real datasets include Zachary’s Karate Club network [31] where we use synthetic opinion vectors, and six Twitter networks from [32, 33] that are gathered with real opinions (computed using sentiment analysis) for different events ranging from political elections to sports. To fit our setting, we process the Twitter networks to make sure that their adjacency matrices are symmetric. See S1 Table for network statistics. Meanwhile, following the way of processing the real opinions in [34], we normalize the first set of opinions for each event into range [0, 1]. After that, we transform the opinions to [−1, 1] for BEBA. The synthetic networks, which are used with randomly generated opinions, are:

- Erdős-Rényi (ER) networks *G*(*n*, *ρ*) with binomial degree distributions, where *ρ* is the edge probability [35];
- Watts-Strogatz (WS) networks *G*(*n*, *K*, *σ*) that have the small world property [36]—with *K* being the average degree and *σ* the rewiring probability;
- Barabási-Albert (BA) networks *G*(*n*, *M*_0_, *M*) that are scale-free, where *M*_0_ is the number of initial nodes and *M* the number of nodes a new node is connected to [37].

### The influence of the entrenchment parameter *β*

From Theorem 2, we know the stationary opinion vector **y** of our model polarizes when $\beta >\frac{1}{{\left[\text{min}|y(0)|\right]}^{2}}$, and reaches consensus when $\beta <\frac{1}{{\left[\text{max}|y(0)|\right]}^{2}}$. These thresholds are far away apart. In practice, the transition between consensus and polarization occurs at a value much lower than $\frac{1}{{\left[\text{min}|y(0)|\right]}^{2}}$ and higher than $\frac{1}{{\left[\text{max}|y(0)|\right]}^{2}}$. We now take the Karate network as an example and examine the relation between *β* and polarization experimentally using random initial opinion vectors.

**Fig 4:**
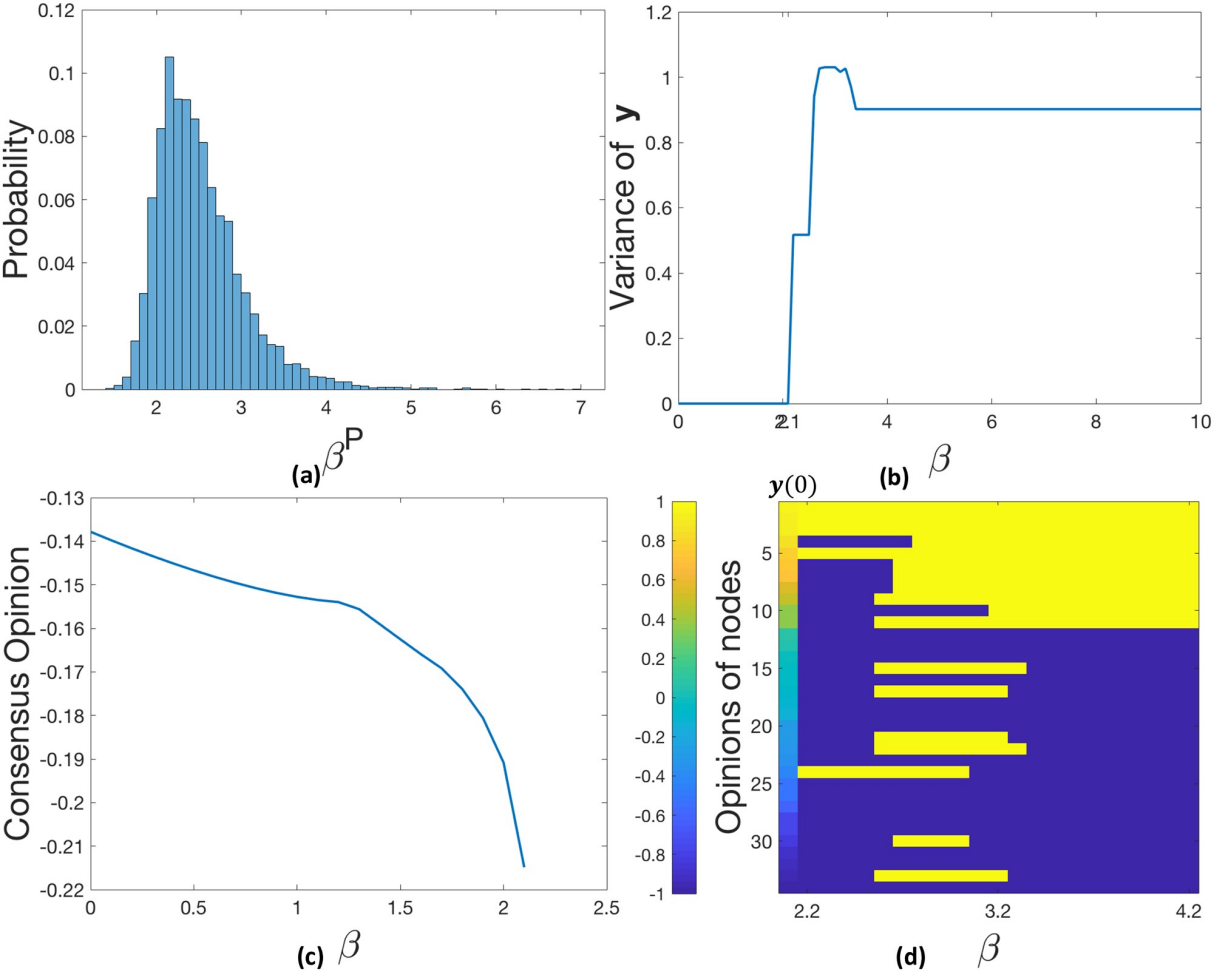
For the Karate network. (a) the distribution of *β*^*P*^ for 10,000 random opinion vectors (uniform on [−1, 1]); For one of the opinion vectors, (b) the variance of all converged **y** as *β* increases from 0 to 10; (c) consensus opinion values for *β* ∈ [0, 2.1]; (d) final converged opinions for each of the nodes.

Let *β*^*P*^ denote the threshold between consensus and polarization for an opinion vector—the smallest *β* that results in polarization. More specifically, what we observe is that consensus is reached when *β* < *β*^*P*^ and the stationary opinions polarize when *β* ≥ *β*^*P*^. Since we do not restrict opinions to be only −*y*_0_ and *y*_0_ as in Corollary 1, there is no persistent disagreement observed in our experiments. Also, note that even though we assume the identical entrenchment parameter for all nodes in a network both in the theoretical and experimental analysis, the chances are people will have different levels of entrenchment in the real world. Fig 4(a) shows the distribution of the empirical *β*^*P*^ values for 10,000 different random opinion vectors, where each opinion is uniformly sampled between [−1, 1]. The value of *β*^*P*^ for each random opinion vector is found by grid search from 0 to 10 at a step size of 0.1. We observe that the threshold for polarization—*β*^*P*^ is much smaller than the theoretical value, which should be around 10^4^ according to the sampled opinions. However, on the Karate network, the empirical value of *β*^*P*^ is below 5 for most of the random **y**(0), and never exceeds 7.

We further study the opinion dynamics for one individual opinion vector from the 10,000 samples. Fig 4(b) shows the variance of its stationary opinions as a function of *β*. We observe that as *β* increases, the opinion vector converges from consensus to polarized states. The variance stays zero if there is consensus, while when the variance is greater than zero, polarization is obtained (i.e., different variances correspond to different polarized states). For this **y**(0), the transition from consensus to polarization happens at *β*^*P*^ = 2.2 and no persistent disagreement was observed.

When consensus is reached, Fig 4(c) shows that the consensus value becomes less neutral as *β* increases. This is true for 78.74% of the 10,000 random opinion vectors on the Karate network. Meanwhile, different values of *β* do not necessarily result in the same polarized state. The heatmap Fig 4(d) shows different polarized states with different values of *β* for this **y**(0), where each column corresponds to a specific value of *β* and each row to a specific node. The color indicates the node opinions with the dark blue being −1 and yellow being 1.

### The influence of the initial opinions y(0)

**Fig 5:**
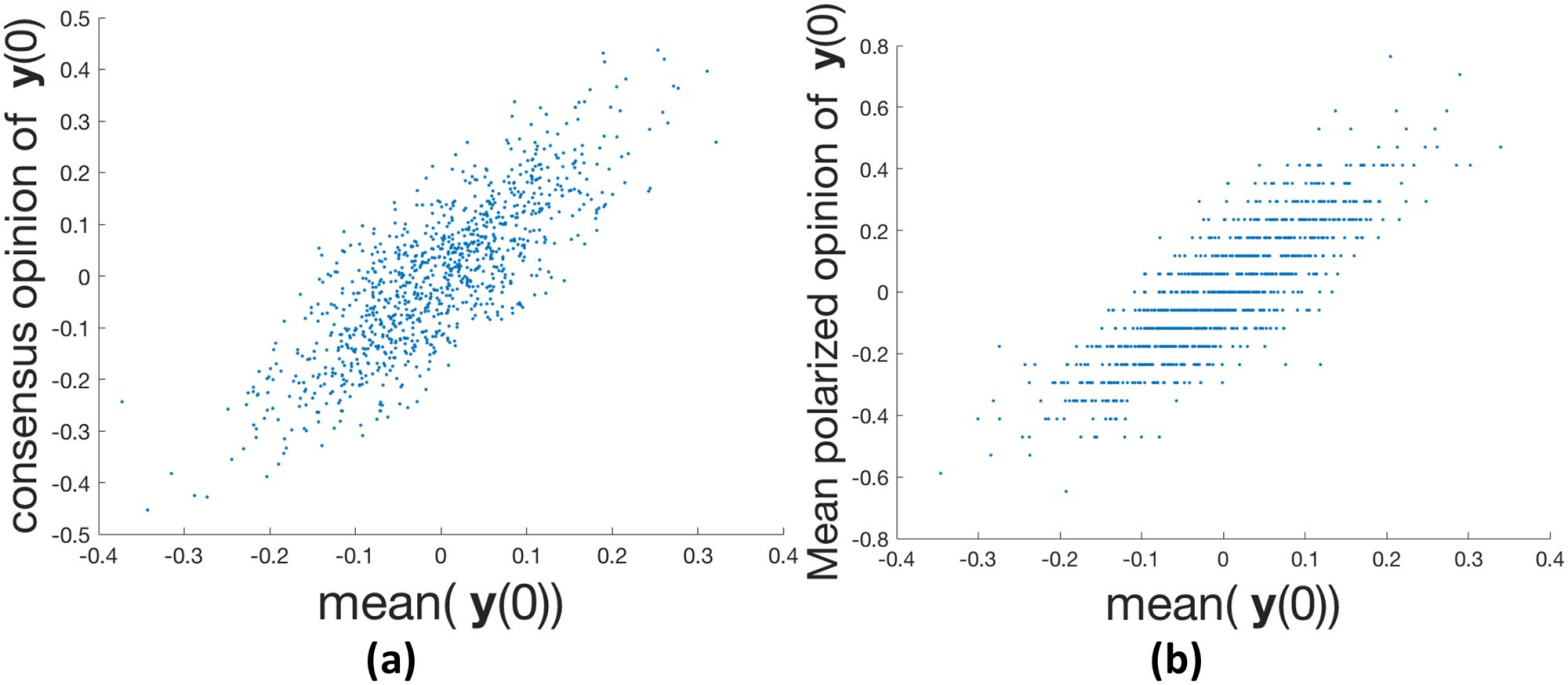
For 1,000 random y(0) on Karate network. (a) consensus opinion when *β* = 1; (b) mean polarized opinion when *β* = 10.

In this experiment, we investigate the influence of **y**(0) on the consensus opinion value and the mean polarized opinion. We observed that the consensus value as well as the mean polarized opinion are strongly correlated with the mean of **y**(0), as shown in Fig 5. Meanwhile, in the case of polarization (Fig 5(b)), opinion vectors with similar initial means may result in quite different polarized states because the placement of the opinions on the graph nodes differs. Also, **y**(0) with different means could result in similar polarized states with the same mean polarized opinion.

We also investigate the influence of the initial opinions on real-world dataset. Tw:Club with real opinions on whether Barcelona was getting the first place in La-liga 2016, and Tw: Sport with opinions on whether Juventus or Real Madrid is winning the Champions League final in 2015, have the same network but different opinion vectors [32], thus suitable for this evaluation. We found that the *β*^*P*^ is 11.7 for Tw:Club and 3.3 for Tw:Sport. The results indicate that the support behavior for different football clubs gets polarized more easily than a single YES/No question. With BEBA, we are able to quantify how easily people’s opinions on an event may get polarized.

### The influence of the network *G*

In this experiment, we study how the network topology affects the *β*^*P*^ value and the stationary opinions of our model. To this end, we generate random networks of the three models with the same number of nodes and similars number of edges, and intialize the same (set of) opinion vectors **y**(0) for them.

**Fig 6:**
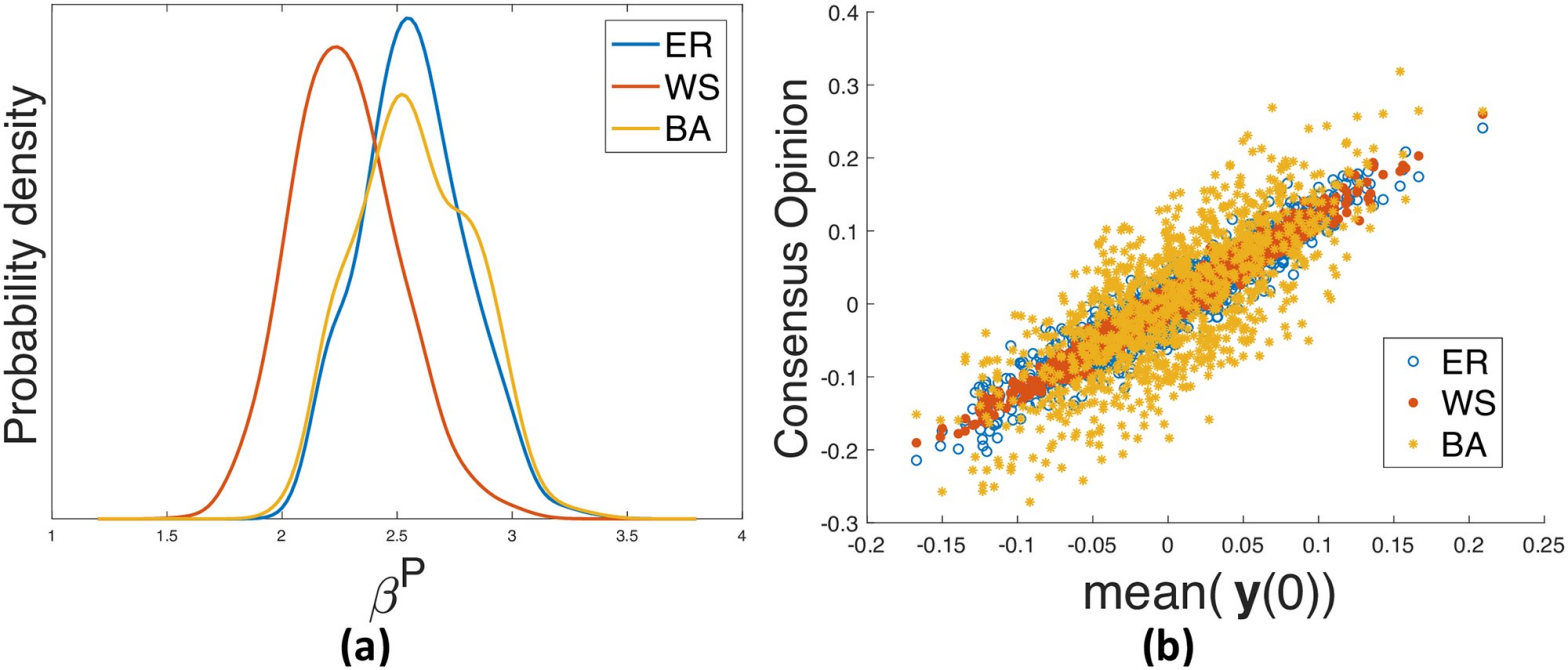
Based on one ER model (*n* = 100, *ρ* = 0.0606), one WS model (*n* = 100, *K* = 6, *σ* = 0.2), and one BA model (*n* = 100, *M*_0_ = 4, *M* = 3). (a) distribution of *β*^*P*^ for 1,000 random opinion vectors; (b) for 1,000 opinion vectors, the relation between the consensus value and the mean **y**(0) when *β* = 1.

**Fig 7:**
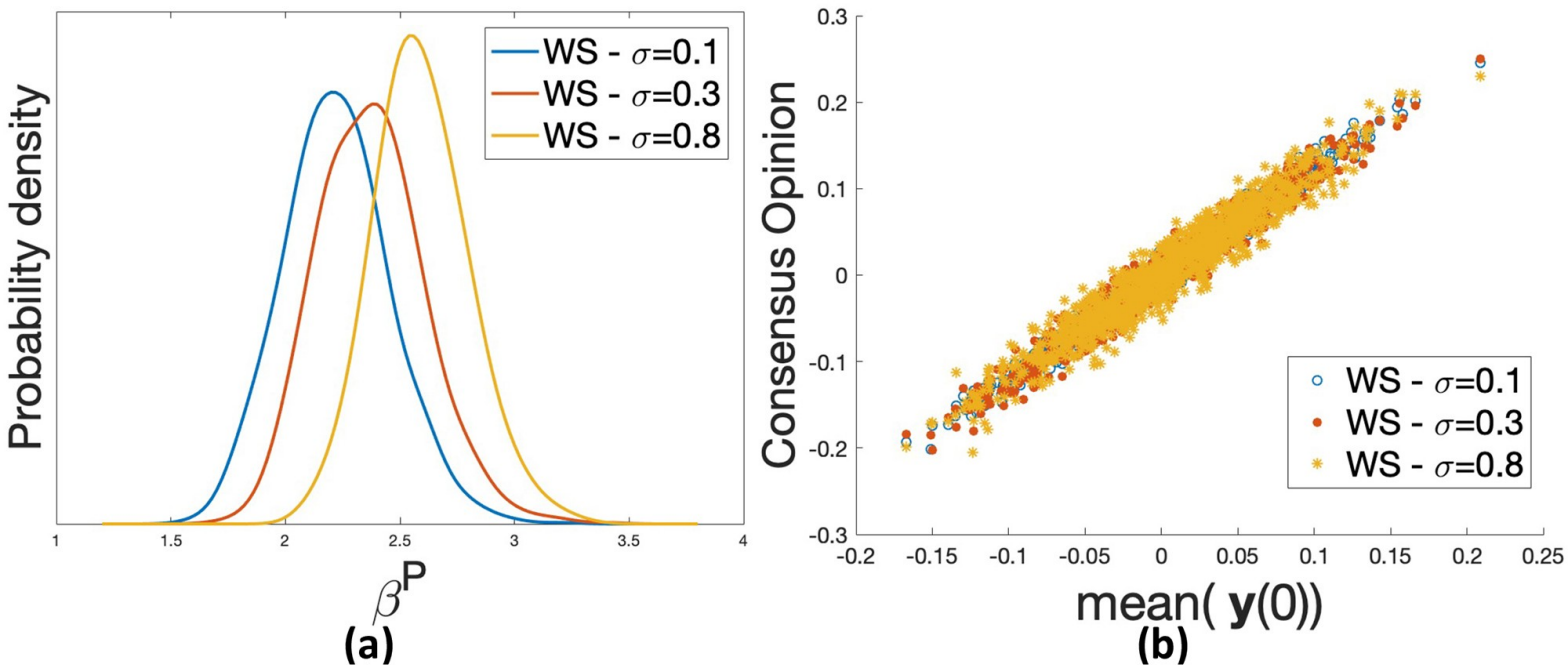
Based on three WS models with different rewiring probabilities (*n* = 100, *K* = 6, *σ* = 0.1, 0.3, 0.8). (a) distribution of *β*^*P*^ for 1,000 random opinion vectors; (b) for 1,000 opinion vectors, the relation between the consensus value and the mean **y**(0) when *β* = 1.

We observe that different network properties result in different dynamics of polarization. Shown in Fig 6(a) are the distributions of the *β*^*P*^ values on the three models for the same set of **y**(0). The BA model has a larger standard deviation of the *β*^*P*^ values, which appears to be due to ‘hub’ nodes whose opinions strongly affect the value of *β*^*P*^. The ER model has similar mean of *β*^*P*^ to the BA model, which is larger than that of the WS model. As the WS model with the rewiring probability 1 essentially approaches the ER model, our WS network with less randomness (i.e., a rewiring probability of 0.2) in Fig 6(a) shows a tendency to get polarized more easily than the ER model. It indicates that, for the same set of opinion vectors on different issues, the more randomness the network has, the more robust the network is against polarization. To further verify this, we do similar experiments with the same set of opinion vectors on the WS models with more rewiring probabilities of 0.1, 0.3, and 0.8, see Fig 7(a). It shows that as the rewiring probability of the WS model increases, the mean of *β*^*P*^ becomes larger, which confirms our observation that the randomness in networks correlates with the networks’ resilience against polarization.

The consensus values reached by the same set of opinion vectors on the three types of networks are plotted in Fig 6(b). The shapes of scatter plots become increasingly compact from the BA model, to the ER model, and then to the WS model, indicating the largest and the smallest variance on the consensus opinions for the BA and the WS network, respectively. The large variance for the BA mode is caused by the ‘hub’ nodes. Comparing to the ER mode, the WS mode here does not have much randomness, thus its consensus opinion varies the least. Fig 7(b) also confirms that the WS model with a smaller rewiring probability (i.e., less randomness) has a more compact shape. Similar to the results shown in Fig 5, we also compare the influence of **y**(0) on three different types of random networks. The finding is consistent with that of Fig 6(b), see S1 Fig.

The placement of the edges and the parameters in each model also affect the opinion dynamics. We take the ER model as the example and investigate the influence of *G* with a fixed and a changing *ρ* for one random opinion vector. On 1,000 ER networks with *ρ* = 0.4, the *β*^*P*^ as well as the consensus opinion for that opinion vector vary, see S2 Fig. If we increase *ρ* from a small value, which still guarantees a connected network, to 1, we observe quite different *β*^*P*^ for that opinion vector even with similar values of *ρ*. While when *ρ* gets closer to 1, meaning that the network gets more connected, that *β*^*P*^ becomes more stable, see S3 Fig. The results are similar for the consensus value, and the polarized opinion.

### Real-world dataset analysis

Using the six real-world twitter datasets [32, 33], we investigate how easily each event gets polarized opinions, namely the value of *β*^*P*^. It is shown in Table 1 that political events concerning elections in the first row (Tw:UK for the British election 2015, Tw:Delhi for the Delhi Assembly election 2013, and Tw:US for the US Presidential election 2016) are less likely to polarize since they require a relatively high *β*^*P*^. However, the 2016 US presidential election shows a tendency to get polarized more easily than the other two elections with a lower *β*^*P*^. On the other hand, the TV (Tw:GoT for the promotion of the TV show ‘Games of Thrones’ in 2015) and sport (Tw:Club) events are more likely to get polarized, except when people have to bet on a result (Tw:Club) instead of supporting.

**Table 1:** *β*^*P*^ for real-world twitter datasets.

Network	*β* ^*P*^	Network	*β* ^*P*^	Network	*β* ^*P*^
Tw:UK	7.5	Tw:Delhi	7.7	Tw:US	4.9
Tw:GoT	2.9	Tw:Club	3.3	Tw:Sport	11.7

### Opinion manipulation under BEBA

We also investigate the following question as a potential application of our model on opinion manipulation: how will the opinion dynamics be influenced by edge addition or deletion in networks? We use the Karate network to study this question experimentally.

We observe that in order to maximally decrease the consensus opinion by editing one edge, adding the edge between the most opinionated disconnected negative nodes is the best choice if allowed a single edge addition; while deleting the edge between the most opinionated connected positive nodes is the best choice if allowed a single edge deletion. Similarly, the maximal decrease of the consensus value can be achieved by adding the edge between the most positively opinionated nodes or deleting the edge between the most negatively opinionated nodes. See S4 and S5 Figs.

Another interesting finding is that the connections between nodes with opposing equivalent opinions (i.e., in terms of absolute value) have almost no influence on the consensus value, see S6 Fig. In contrast, when the network gets polarized, the neighbors of the neutral nodes have more significant influence on the mean polarized opinions.

## Conclusion and future work

Modeling how opinions evolve when individuals interact in social networks is an important computational social science challenge that has received renewed attention recently. The availability of realistic models of this type may have substantial real-life impact on a variety of applications, from political campaign design, to conflict prevention and mitigation. A large number of models have been proposed in the literature towards this end. To the best of our knowledge, however, none of them model the so-called Backfire Effect: the fact that individuals, when exposed to a strongly opposing view, will not be moderated, but rather become more entrenched in their opinion.

Here we proposed the BEBA model, which models both Biased Assimilation and Backfire Effect. It is governed by one parameter (which can vary over the individuals), called the entrenchment parameter, determining the strength of both. The BEBA model naturally generates different behaviors: from convergence to a consensus, to polarization. Theoretical and empirical analyses demonstrate that the resulting model is not only practical, its behavior also provides an interesting view on the interplay between network structure, the entrenchment parameter, and the opinions.

These properties make BEBA a useful tool for simulating the effect of interventions, such as editing the network (e.g. by facilitating communication between particular pairs of individuals), altering the initial opinions (e.g. through targeted information campaigns), or affecting the entrenchment of particular individuals (e.g. through education). It has the potential to help with correcting the misinformation in the real world.

However, BEBA has its limitations. For example, it would be interesting to investigate a variant of the model where the updated opinions naturally fall into the range [−1, 1] without the clipping we applied in Eq (5). Also, it would be interesting to explore the different parameters for the Backfire Effect and Biased Assimilation We plan to explore these directions in the future.

## Supporting information

S1 Appendix Proof of Theorem 2. This proof includes two cases: only one node in the environment, and a group of nodes in the environment.
(PDF)

S2 Appendix Proof of Theorem 2. (PDF)

S1 Table Real-world network summary. (PDF)

S1 Fig For 1,000 random y(0). (a) and (b) on a BA model (*n* = 34, *M*_0_ = 3, *M* = 2); (c) and (d) on an ER model (*n* = 34, *ρ* = 0.139); (e) and (f) on a WS model (*n* = 34, *K* = 4, *σ* = 0.2). The left column of (a), (c), (e)—the relation between the consensus opinion and the mean **y**(0) when *β* = 1; the right column of (b), (d), (f)—the relation between the mean polarized opinion and the mean **y**(0) when *β* = 10.
(TIF)

S2 Fig For a random opinion vector y(0) with mean −0.0395, on 1,000 ER models with *n* = 100 and *ρ* = 0.4. (a) the value of *β*^*P*^ for that **y**(0); (b) the consensus opinion reach by **y**(0) when *β* = 1.
(TIF)

S3 Fig For a random opinion vector y(0), on ER models with *n* = 100 and *ρ* ∈ (0,1]. (a) the value of *β*^*P*^ for that **y**(0); (b) the consensus opinion reach by **y**(0) when *β* = 1.
(TIF)

S4 Fig Add one edge on Karate network to change the consensus opinion—*β* = 1. Top 10 best choices are highlighted: green for increase and red for decrease.
(TIF)

S5 Fig Delete one edge on Karate network to change the consensus opinion—*β* = 1. Top 5 best choices are highlighted: green for increase and red for decrease.
(TIF)

S6 Fig Influence of edge edition on consensus. (a) Additions and (b) Deletions that cause minor change (i.e., <10^−3^) in consensus values on Karate network.
(TIF)
