# Supplementary material for: Opinion dynamics with backfire effect and biased assimilation
Source: PLoS One. 2021 Sep 1;16(9):e0256922. doi: 10.1371/journal.pone.0256922 (PMC8409649; doi:10.1371/journal.pone.0256922)
Supplement: S1 Appendix — This proof includes two cases: only one node in the environment, and a group of nodes in the environment. (PDF) [file pone.0256922.s001.pdf]

Proof of Theorem 1

Only one node in the environment

Recall that there is one node with a fixed opinion $p \in [-1, 1]$ in the environment. The opinion of the agent is updated as mentioned in Eq. (6),

Lemma 3. *If $w + \beta p y(t) + 1 \leq 0$, the opinion of the agent stays at $\text{sgn}(y(t))$ for all $t' > t$.*

Proof. As shown in the updating rule that when $w + \beta p y(t) + 1 \leq 0$, $y(t+1) = \text{sgn}(y(t))$. $w + \beta p y(t) + 1 \leq 0$ is equivalent to $\beta p y(t) \leq -w - 1 < 0$. Knowing that $|y(t+1)| = 1 \geq |y(t)|$,

$$\beta p y(t+1) \leq -w - 1$$

Therefore, $y(t') = \text{sgn}(y(t+1)) = \text{sgn}(y(t))$ for all $t' > t$. □

Lemma 4. *If $w + \beta p y(t) + 1 > 0$, there exist two fixed points where $y(t+1) = y(t)$: p and $-\frac{1}{\beta p}$. p is attracting while $-\frac{1}{\beta p}$ is repelling.*

Proof. The converged opinion y of the agent should satisfy

$$f(y) = \frac{wy + \beta p^2 y + p}{w + \beta p y + 1}$$

$$f(y) - y = \frac{-\beta p y^2 + (\beta p^2 - 1)y + p}{w + \beta p y + 1} = \frac{u(y)}{v(y)} = 0 \tag{7}$$

where

$$u(y) = -\beta p y^2 + (\beta p^2 - 1)y + p$$
$$v(y) = \beta p y + w + 1$$

By solving $u(y) = 0$, which is equivalent to $f(y) - y = 0$ since $u(y) > 0$, the two fixed points of $f(y)$ are: p and $-\frac{1}{\beta p}$.

Next, we prove that p is attracting and $-\frac{1}{\beta p}$ is repelling.

$$f'(y) = \frac{w(w + \beta p^2)}{(w + \beta p y + 1)^2} \geq 0$$

$|f'(y)| = f'(y)$, then $f'(p) = \frac{w(w+\beta p^2)}{(w+\beta p^2+1)^2} < 1$, thus attracting; while $f'(y - \frac{1}{\beta p}) = \frac{w(w+\beta p^2)}{w^2} > 1$, thus repelling. □

Lemma 5. *If $w + \beta p y(t) + 1 > 0$ and $p y(t) \geq 0$, $y = p$.*

Proof. If $p = 0$, $y(t+1) = \frac{w}{w+1} y(t)$, as the iteration goes, $\lim_{t\to\infty} y(t) = 0$;

If $p y(t) > 0$, e.g., they are both positive

- when $0 < y(t) < p$, $y(t+1) - y(t) = \frac{u(y(t))}{v(y(t))} > 0$, thus $y(t+1) > y(t)$, the agent's opinion increases until it reaches p;
- when $p < y(t) < 1$, $y(t+1) - y(t) < 0$, the agent's opinion decreases to p.

□

Lemma 6. *If $w + \beta p y(t) + 1 > 0$ and $p y(t) < 0$,*

1. *If* $\left|\frac{1}{\beta p}\right| > 1$, $\lim_{t\to\infty} y(t) = y^e$.

2. *If* $\left|\frac{1}{\beta p}\right| \leq 1$,

   (a) *If* $|y(t)| < \left|\frac{1}{\beta p}\right|$, $y = p$.

   (b) *If* $y(t) = -\frac{1}{\beta p}$, $y(t') = -\frac{1}{\beta p}$ *for all* $t' \geq t$.

   (c) *If* $\left|\frac{1}{\beta p}\right| < |y(t)| \leq 1$, $y = \mathrm{sgn}(y(t))$.

Proof. Assume $y(t) \in (0, 1]$ and $p \in (-1, 0)$,

- if $\left|\frac{1}{\beta p}\right| > 1$, all $y(t) \in (0, 1] < -\frac{1}{\beta p}$, $y(t)$ is attracted to p as the updating goes;
- if $\left|\frac{1}{\beta p}\right| = 1$, $y(t)$ is repelled by the extreme point and goes to the attracting one unless it starts with $-\frac{1}{\beta p}$ at time t;
- if $\left|\frac{1}{\beta p}\right| < 1$, when $0 < y(t) < -\frac{1}{\beta p}$, $y(t+1) - y(t) = \frac{u(y(t))}{v(y(t))} < 0$, $y(t+1) < y(t)$, the agent's opinion decreases to p; when $y(t) = -\frac{1}{\beta p}$, $y(t)$ stays there; when $y(t) > -\frac{1}{\beta p}$, $y(t+1) > y(t)$, the agent's opinion increases to the extreme value on its side.

□

A group of nodes in the environment

Assume there is a set of m neighbour with different fixed opinions, $\mathbf{p} = (p_1, p_2, ..., p_m)$, $m > 1$. We denote

- $q = \sum_j p_j^2$ the sum of the squares of the fixed opinions.
- $s = \sum_j p_j$ the sum of the fixed opinions.
- $m = \sum_j 1$ the number of nodes in the environment.

Lemma 7. *$mq - s^2 \geq 0$, which is $m \sum_j p_j^2 \geq (\sum_j p_j)^2$.*

Proof.

$$m \sum_j p_j^2 - (\sum_j x_j)^2 = \frac{1}{2} \sum_i \sum_j (p_i - p_j)^2 \geq 0$$

□

The agent's opinion is updated by

$$y(t+1) = \begin{cases} \mathrm{sgn}(y(t)) & \text{if } w + \beta s y(t) + m \leq 0, \\ \frac{w y(t) + \beta q y(t) + s}{w + \beta s y(t) + m} & \text{otherwise.} \end{cases} \tag{8}$$

Lemma 8. *If $w + \beta s y(t) + m > 0$, there exist two fixed points where $y(t+1) = y(t)$:*

$$y^a = \frac{\beta q - m + \sqrt{\Delta}}{2\beta s} \qquad y^r = \frac{\beta q - m - \sqrt{\Delta}}{2\beta s}$$

where $\Delta = (\beta q - m)^2 + 4\beta s^2$. y^a is attracting while y^r is repelling.

Proof. The function is $f(y) = \frac{wy+\beta qy+s}{w+\beta sy+m}$. The two fixed points satisfy $f(y) = y$. $|f'(y)| = f'(y)$ since

$$\begin{aligned} f'(y) =& \frac{(w+\beta q)(w+m) - \beta s^2}{(\beta sy + w + m)^2} \\ =& \frac{w(w+m) + \beta qw + \beta(qm - s^2)}{(\beta sy + w + m)^2} > 0 \end{aligned}$$

For $y^a = \frac{\beta q - m + \sqrt{\Delta}}{2\beta s}$, $f'(y^a) < 1$ because

$$\begin{aligned} f'(y^a) - 1 =& -\frac{1}{2}\frac{(m-\beta q)^2 + 4\beta s^2 + (2w+m+\beta q)\sqrt{\Delta}}{(\beta sy^a + w + m)^2} \\ <& 0 \end{aligned}$$

For $y^r = \frac{\beta q - m - \sqrt{\Delta}}{2\beta s}$, $f'(y^r) > 1$ because

$$\begin{aligned} f'(y^r) - 1 =& -\frac{1}{2}\frac{(m-\beta q)^2 + 4\beta s^2 - (2w+m+\beta q)\sqrt{\Delta}}{(\beta sy^r + w + m)^2} \\ =& -\frac{1}{2}\frac{A}{B} \end{aligned}$$

$\frac{A}{B} < 0$ since $B > 0$ and it can be proved as below that $A < 0$.

$$\begin{aligned} & \left[(m-\beta q)^2 + 4\beta s^2\right]^2 - \left[(2w+m+\beta q)\sqrt{\Delta}\right]^2 \\ =& 4\left[(m-\beta q)^2 + 4\beta s^2\right]\left[\beta(s^2 - qm) - w(m+w+\beta q)\right] \\ <& 0 \end{aligned}$$

Therefore, y^a is attracting and y^r is repelling. □
