# Supplementary material for: Opinion dynamics with backfire effect and biased assimilation
Source: PLoS One. 2021 Sep 1;16(9):e0256922. doi: 10.1371/journal.pone.0256922 (PMC8409649; doi:10.1371/journal.pone.0256922)
Supplement: S2 Appendix — (PDF) [file pone.0256922.s002.pdf]

Proof of Theorem 2

Recall that $y_i(t) \in (-1, 0) \cup (0, 1)$. Given any opinion vector $\mathbf{y}(0)$ of a given connected network $G = (V, E)$, the opinions can be divided into two groups V_1 and V_2 at any time t: a) $\forall i \in V_1$, $y_i(t) > 0$; b)$\forall i \in V_2$, $y_i(t) < 0$, and $V = V_1 \cup V_2$. Denote $n_i^s(t)$ the number of node i's neighbors node that are in the **s**ame group with i at time t, and $n_i^d(t)$ the number of neighbors in the **d**ifferent group. Specifically, they are denoted as

$$\begin{aligned} n_i^s(t) =& |N(i)^s|, N(i)^s = \{j | j \in N(i), \text{ and } y_i(t)y_j(t) > 0\} \\ n_i^d(t) =& |N(i)^d|, N(i)^d = \{k | k \in N(i), \text{ and } y_i(t)y_k(t) < 0\} \end{aligned}$$

Lemma 9. *For node $i \in V$ fix $\beta_i = \beta > 0$, if $\beta > \frac{1}{[\min(|\mathbf{y}(0)|)]^2}$, $\lim_{t\to\infty} |y_i(t)| = 1$.*

Proof. For node $i \in V$, the opinion is updated with BEBA. If $\gamma = 1 + \sum_{j \in N(i)} w_{ij} \leq 0$, $y_i(t+1)$ reaches the extreme value in one iteration due to strong backfire effect.

While when $\gamma > 0$, for any $t > 0$, $y_i(t+1)$ is updated as

$$y_i(t) \frac{1 + \sum_{j \in N(i)^s} w_{ij} \frac{y_j(t)}{y_i(t)} + \sum_{k \in N(i)^d} w_{ik} \frac{y_k(t)}{y_i(t)}}{1 + \sum_{j \in N(i)^s} w_{ij} + \sum_{k \in N(i)^d} w_{ik}} = y_i(t) \frac{C}{D} \tag{9}$$

When $\beta > \frac{1}{[\min(|\mathbf{y}(t)|)]^2}$, for all $k \in N(i)^d$, $w_{ik} = \beta y_i(t) y_k(t) + 1 < 0$. The sums in Eq. (9) satisfy: $\sum_{j \in N(i)^s} w_{ij} \frac{y_j(t)}{y_i(t)}$, $\sum_{j \in N(i)^s} w_{ij}$, $\sum_{k \in N(i)^d} w_{ik} \frac{y_k(t)}{y_i(t)} > 0$, and $\sum_{k \in N(i)^d} w_{ik} < 0$.

Now we focus on the node that has the most moderate opinion, namely the node with absolute value of opinion $\min|\mathbf{y}(t)|$ at each time step, starting from time 0. Knowing $C, D > 0$,

$$C - D = \sum_{j \in N(i)^s} w_{ij} \left(\frac{y_j(t)}{y_i(t)} - 1\right) + \sum_{k \in N(i)^d} w_{ik} \left(\frac{y_k(t)}{y_i(t)} - 1\right) \tag{10}$$

Since $y_i(t)$ has the smallest absolute opinion value, for any $j \in N(i)^s$, $\frac{y_j(t)}{y_i(t)} \geq 1$, thus $C > D$, $\frac{C}{D} > 1$, and $|y_i(t+1)| > |y_i(t)|$.

After every iteration from time t to $t+1$, the opinion of the most moderate node becomes more extreme, until it reaches the absolute value of 1, thus for any $i \in V$, $\lim_{t\to\infty} |y_i(t)| = 1$. ◻

Lemma 10. *For node $i \in V$, if $\beta < \frac{1}{[\max(|\mathbf{y}(0)|)]^2}$, there exists a unique $y^* \in [-\max(|\mathbf{y}(0)|), \max(|\mathbf{y}(0)|)]$ such that $\lim_{t\to\infty} y_i(t) = y^*$ for all $i \in V$.*

Proof. When $\beta < \frac{1}{[\max(|\mathbf{y}(0)|)]^2}$, $\gamma = 1 + \sum_{j \in N(i)} w_{ij} > 0$ because for any $j \in N(i)$, $w_{ij} = \beta y_i(t) y_j(t) + 1 > 0$.

For any $t > 1$, $y_i(t+1)$ is updated as in Eq. (9), however, the sums have different values: $\sum_{j \in N(i)^s} w_{ij} \frac{y_j(t)}{y_i(t)}$, $\sum_{j \in N(i)^s} w_{ij}$, $\sum_{k \in N(i)^d} w_{ik} > 0$, and $\sum_{k \in N(i)^d} w_{ik} \frac{y_k(t)}{y_i(t)} < 0$.

Then we focus on the most opinionated node, which means the node has the largest absolution value of its opinion $\max|\mathbf{y}(t)|$, starting from time 0. Knowing $D > 0$,

- when $C > 0$, $C - D$ is shown in Eq. (10). With i being the most opinionated node, $\frac{y_j(t)}{y_i(t)} \leq 1$ for all $j \in N(i)^s$; $\frac{y_k(t)}{y_i(t)} < 0$ for all $k \in N(i)^d$. Therefore, $C < D$, $0 < \frac{C}{D} < 1$ and $|y_i(t+1)| < |y_i(t)|$.
- when $C = 0$, $y_i(t+1) = 0$.
- when $C < 0$, $-C - D$ is shown in Eq. (11). As $-1 \leq \frac{y_k(t)}{y_i(t)} \leq 0$ for $k \in N(i)^d$, $-C - D < 0$, $0 < \left|\frac{C}{D}\right| < 1$, thus $|y_i(t+1)| < |y_i(t)|$.

$$-2-\sum_{j\in N(i)^s} w_{ij}(\frac{y_j(t)}{y_i(t)}+1)-\sum_{k\in N(i)^d} w_{ik}(\frac{y_k(t)}{y_i(t)}+1) \tag{11}$$

At every time step, the most opinionated node get moderated until they reach consensus - there is no such node and the updating process stops because consensus is reached.

□

Lemma 11. *For node $i \in V_1$, $y_i(0) = y_0$, where $0 < y_0 < 1$; $\forall i \in V_2$, $y_i(0) = -y_0$. If $\beta = \frac{1}{y_0^2}$, $y_i(t) = y_i(0)$ for all $t \geq 0$.*

Proof. When $\beta = \frac{1}{y_0^2}$, $w_{ij} = \frac{1}{y_0^2} y_i(t)y_j(t)$. At time 1,

$$y_i(1) = \frac{y_i(0) + 2n_i^s(0)y_i(0)}{1 + 2n_i^s(0)} = y_i(0)$$

For any $t \geq 1$,

$$y_i(t+1) = \frac{y_i(t) + 2n_i^s(t)y_i(t)}{1 + 2n_i^s(t)} = y_i(t) = y_i(0)$$

□
