# Supplementary material for: Opinion dynamics with backfire effect and biased assimilation
Source: PLoS One. 2021 Sep 1;16(9):e0256922. doi: 10.1371/journal.pone.0256922 (PMC8409649; doi:10.1371/journal.pone.0256922)
Supplement: S1 Table — (PDF) [file pone.0256922.s003.pdf]

Table 2. Real-world Network Summary

Network	$\|V\|$	$\|E\|$	Event
Karate	34	78	Friendship
Tw:Club	703	3322	Barcelona in La-liga 2016
Tw:Sport	703	3322	Juventus vs Real Madrid 2015
Tw:US	533	13564	US Presidential Election 2016
Tw:UK	231	905	British Election 2015
Tw:Delhi	548	3638	Delhi Assembly Election 2013
Tw:GoT	947	7922	GoT promotion 2015
