# Supplementary figures and images for: Opinion dynamics with backfire effect and biased assimilation
Source: PLoS One. 2021 Sep 1;16(9):e0256922. doi: 10.1371/journal.pone.0256922 (PMC8409649; doi:10.1371/journal.pone.0256922)

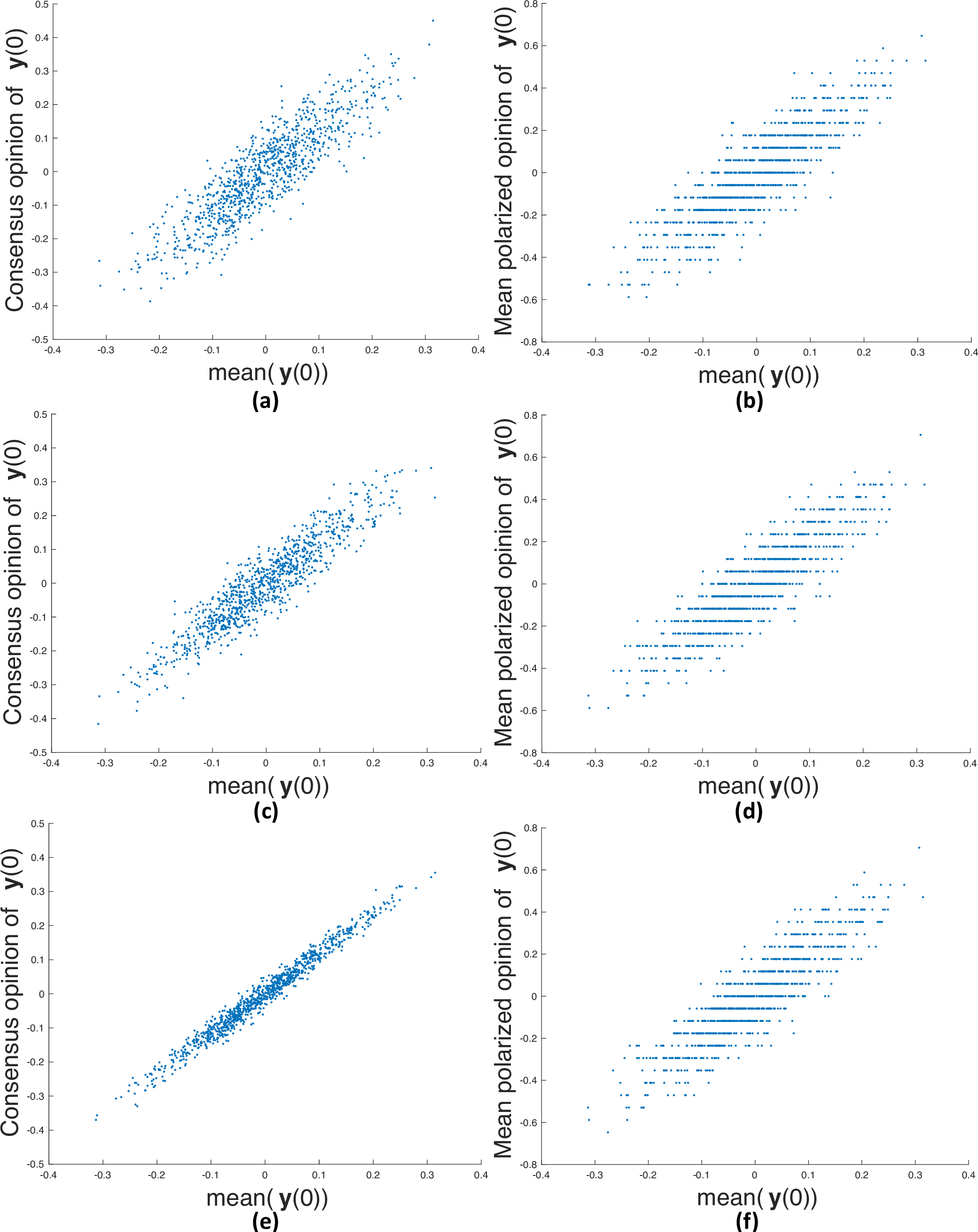

Supplement: S1 Fig — (a) and (b) on a BA model (n = 34, M0 = 3, M = 2); (c) and (d) on an ER model (n = 34, ρ = 0.139); (e) and (f) on a WS model (n = 34, K = 4, σ = 0.2). The left column of (a), (c), (e)—the relation between the consensus opinion and the mean y(0) when β = 1; the right column of (b), (d), (f)—the relation between the mean polarized opinion and the mean y(0) when β = 10. (TIF) [file pone.0256922.s004.tif]

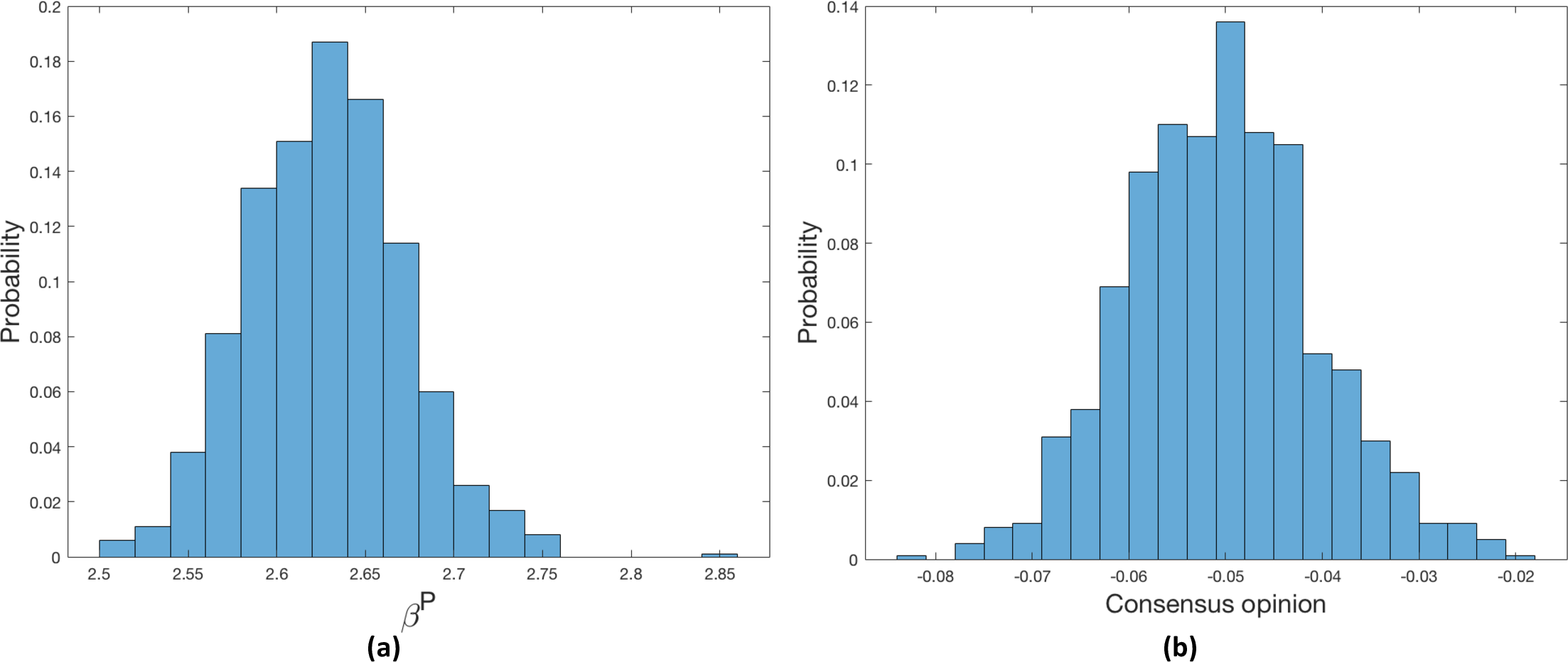

Supplement: S2 Fig — (a) the value of βP for that y(0); (b) the consensus opinion reach by y(0) when β = 1. (TIF) [file pone.0256922.s005.tif]

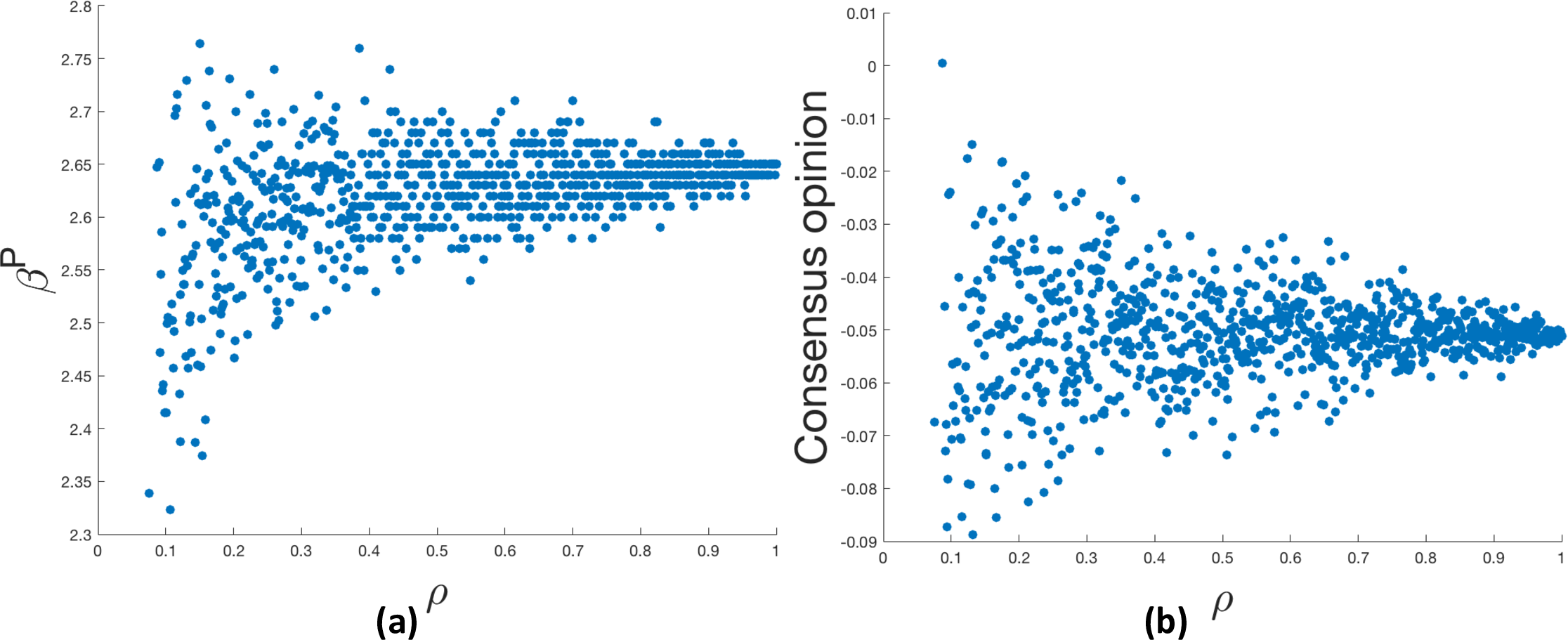

Supplement: S3 Fig — (a) the value of βP for that y(0); (b) the consensus opinion reach by y(0) when β = 1. (TIF) [file pone.0256922.s006.tif]

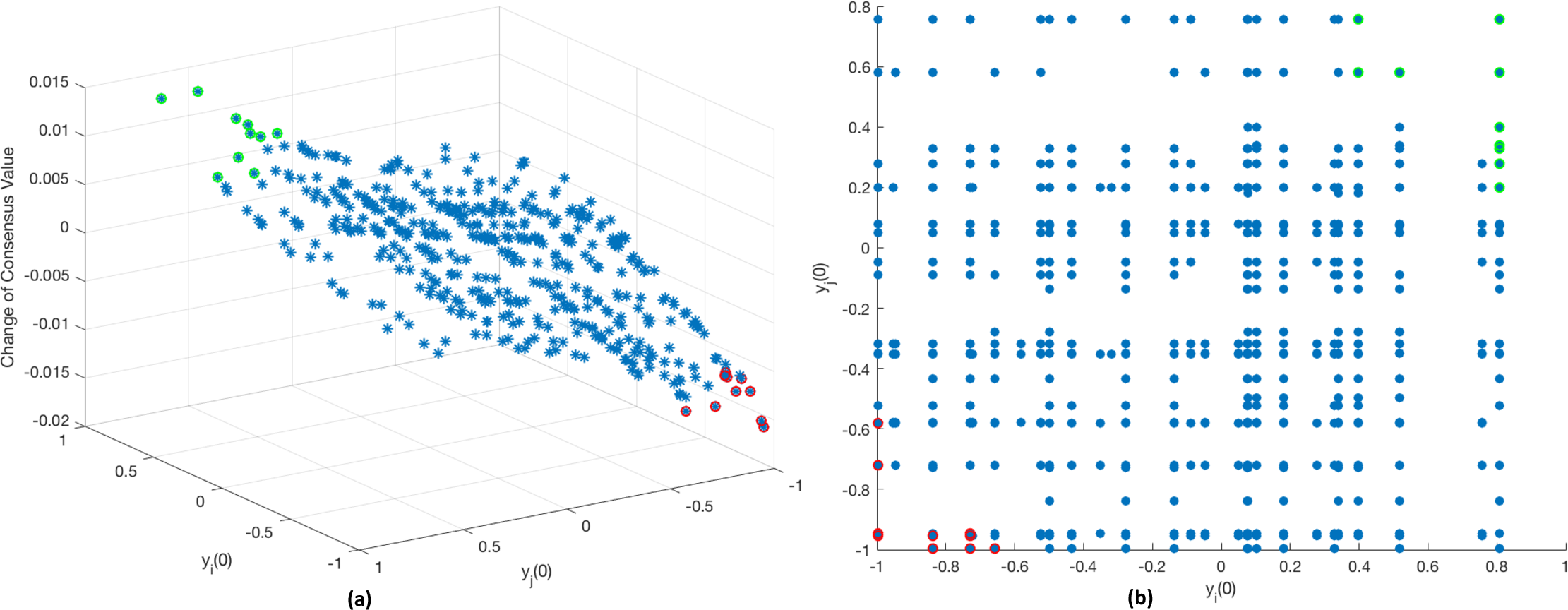

Supplement: S4 Fig — Top 10 best choices are highlighted: green for increase and red for decrease. (TIF) [file pone.0256922.s007.tif]

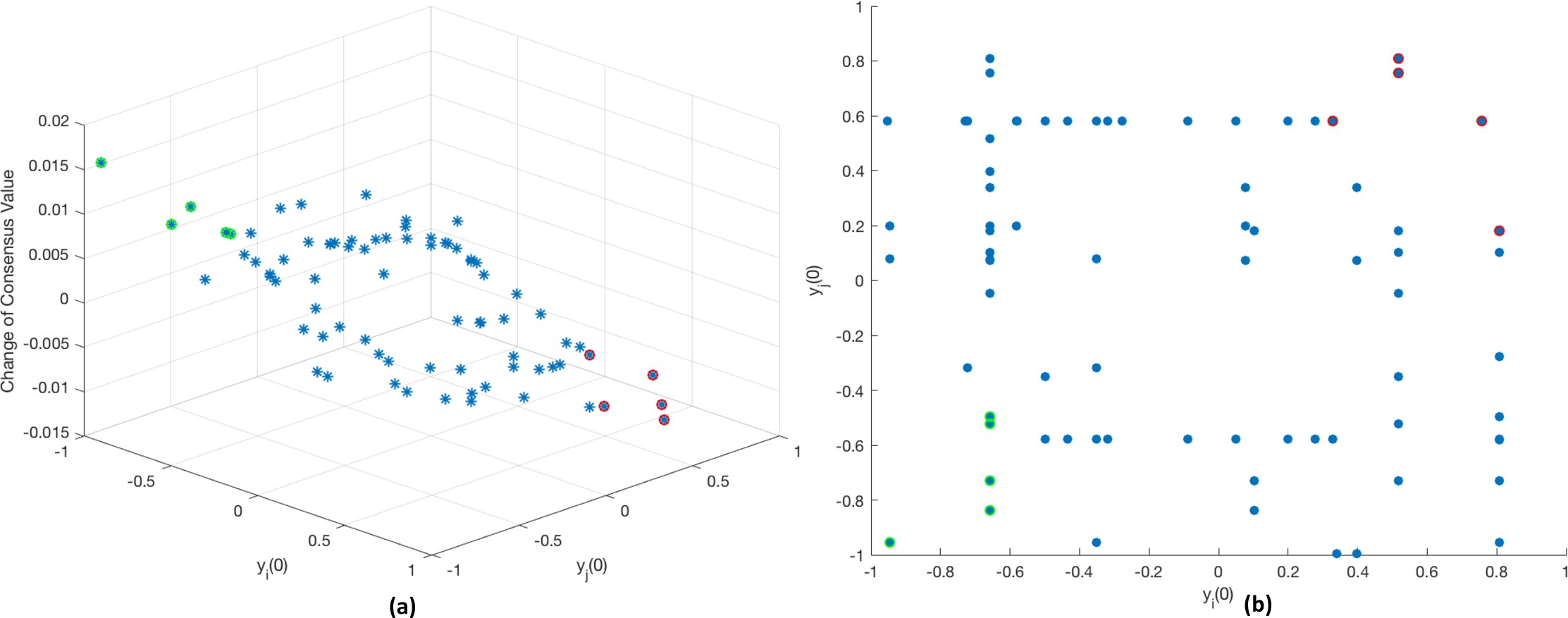

Supplement: S5 Fig — Top 5 best choices are highlighted: green for increase and red for decrease. (TIF) [file pone.0256922.s008.tif]

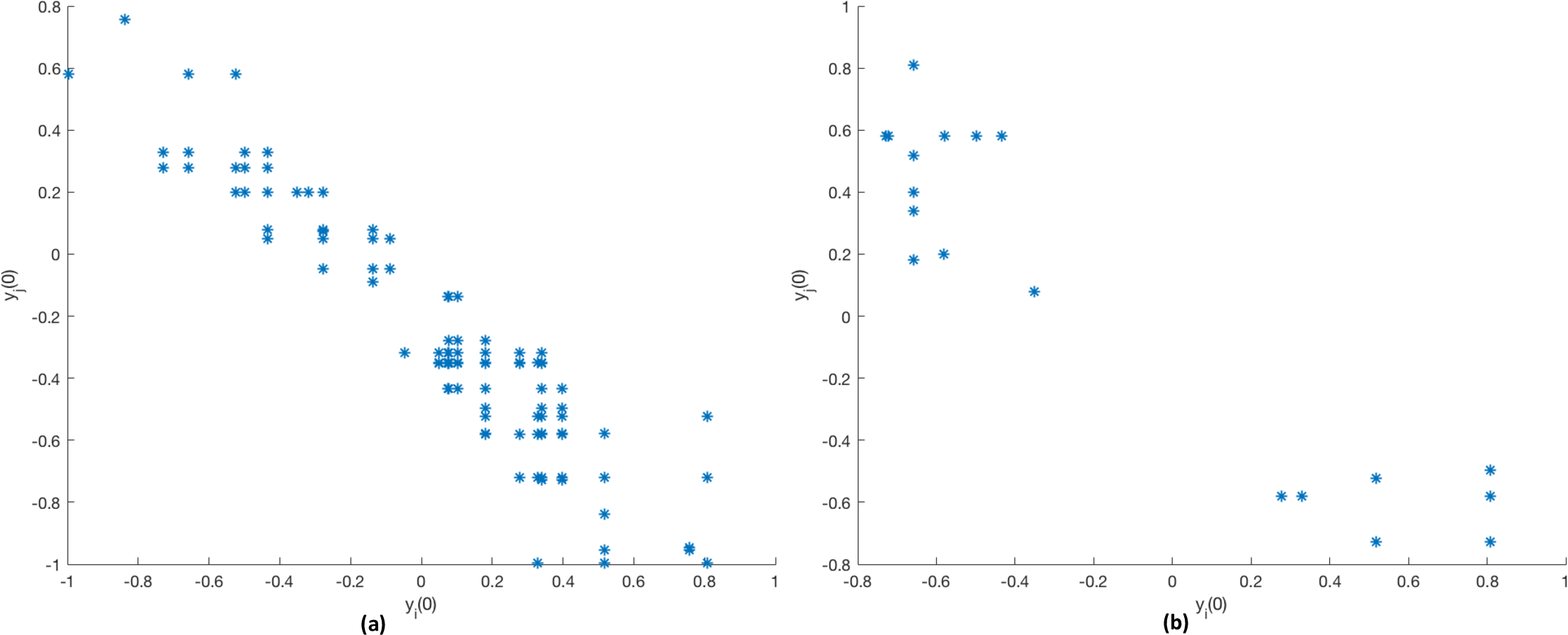

Supplement: S6 Fig — (a) Additions and (b) Deletions that cause minor change (i.e., <10−3) in consensus values on Karate network. (TIF) [file pone.0256922.s009.tif]
